# Editorial: Novel Therapies for Combating Bone Diseases Through Advances in Bone Remodeling

**DOI:** 10.3389/fcell.2021.766963

**Published:** 2021-10-14

**Authors:** Yurui Jiao, Jiake Xu, Chao Liang, Changjun Li

**Affiliations:** ^1^Department of Endocrinology, Endocrinology Research Center, Xiangya Hospital, Central South University, Changsha, China; ^2^School of Biomedical Sciences, The University of Western Australia, Perth, WA, Australia; ^3^School of Chinese Medicine, Law Sau Fai Institute for Advancing Translational Medicine in Bone and Joint Diseases, Hong Kong Baptist University, Hong Kong, Hong Kong, SAR China; ^4^Department of Biology, Southern University of Science and Technology, Shenzhen, China

**Keywords:** bone diseases, bone remodeling, bone cells, molecular targets, translational medicine

Bone is a metabolically active organ that undergoes a constant and continuous state of remodeling throughout life, which is important for the maintenance of normal skeletal structure and function (Salhotra et al., 2020). The coordinated action between bone cells, which are composed of osteoblasts, osteoclasts, and osteocytes, is known as bone remodeling, and the imbalance between the functioning of these cells leads to bone diseases such as osteoporosis, osteoarthritis, rheumatoid arthritis, and bone tumors (Kular et al., 2012; Chen et al.). Bone marrow mesenchymal stem cells (BMSCs), bone-derived exosomes, and microRNAs (miRNAs), which are involved in the regulation of skeletal metabolism, bone remodeling, and bone diseases, have been the current focus of research in the world (Mori et al., 2019; Kalluri and LeBleu, 2020). In recent years, an increasing number of studies were enriched in the area of bone remodeling by researchers, and the most results were encouraging (Li et al., 2015, 2018). These developments, which will provide theoretical advances on bone remodeling and have a growing impact on the treatment of bone diseases in the coming years.

This editorial paper collects 9 publications aimed to explore recent developments with a focus on identifying molecular mechanisms and targets in bone remodeling as well as new therapies for bone disease and associated complications.

## Bone Marrow Mesenchymal Stem Cells

BMSCs display increased adipogenic with age, along with decreased osteogenic differentiation capacity. Based on this theory, one study, Peng et al. identified a differentially expressed ASPH gene, which regulates Wnt signaling mediated by Gsk3β, in middle-aged and elderly aged groups. The depletion of mouse *Asph* suppressed the capacity of osteogenic differentiation and accelerated cellular senescence in BMSCs, while the overexpression of *Asph* enhanced the capacity of osteogenic differentiation and inhibited cellular senescence. Because of the abundant expression of ASPH in a variety of malignant tumors, ASPH has been thought to be a potential therapeutic target for different cancers. Thus, they suggested that the treatment of ASPH inhibitor in patients with cancers needs to be concerned because of their potential risks of bone loss or bone fracture. In a second study, Li et al. explored the effect of calcitonin gene-related peptide (CGRP) on the osteogenic and adipogenic differentiation potential of BMSC. In this study, they indicated that CGRP promoted the osteogenic differentiation of BMSCs while inhibiting their adipogenic differentiation. Aged and ovariectomized mice treated with CGRP showed a substantial promotion of bone formation and a reduction in fat accumulation in the bone marrow. In another study, Xie et al. summarized the significance of Yes-associated protein 1 (YAP1) in orthopedic degenerative diseases. YAP1 can regulate the osteogenic differentiation of BMSCs, as well as the activity of osteoblasts and osteoclastogenesis. Therefore, the regulation of ASPH, CGRP, and YAP1 activity is expected to become a potential intervention strategy to delay the occurrence and development of skeletal degenerative diseases.

## Bone-Derived Exosomes and Mirnas

Emerging numbers of studies have reported miRNAs as important regulators for bone metabolism. Yin et al. focused on the function and mechanism of miR-129-5p in bone metabolism recently and found that the expression of miR-129-5p was enhanced in both aging and menopause osteoporosis models. Overexpression and down-regulation of miR-129-5p respectively inhibited or enhanced osteoblasts differentiation and bone formation by regulating downstream transcription factors of the Wnt/β-catenin pathway through targeting *Tcf4*. Moreover, bioengineered novel recombinant miR-129-5p inhibitor showed a rescue effect on osteoporosis.

Bone-derived exosomes are involved in the regulation of skeletal metabolism, bone remodeling, and pathological processes through modulating intercellular communication and the transfer of materials (Zhu et al., 2018). The founding of Lu et al. suggested that BMSC-derived exosomal miR-29a regulates angiogenesis and osteogenesis, and miR-29a-loaded BMSCs-Exosomes may serve as a robust and potential therapeutic target for osteoporosis. Besides, Lyu et al. and Liu et al. reviewed the current knowledge of exosomes and highlight the application studies of bone-derived exosomes in bone remolding and bone disorders. They summarized the role of exosomes derived from BMSCs, osteoclasts, osteoblasts, and osteocytes in skeletal metabolism, including miR-27a, miR-206a, miR-196a, miR-214, miR-30d-5p, miR-133b-3p, miR-140-3p, miR-140-5p, miR-335-3p, miR-378b, miR-218, miR-1192, miR-680, miR-302a, miR-92a-3b, miR-135b, and miR-100-5p.

## Osteoclasts

The interaction of receptor activator of nuclear factor-kB ligand (RANKL) and its receptor RANK is one of the fundamentals in bone remodeling. The RANK/RANKL system is well-known for regulating bone turnover by promoting the differentiation and activation of osteoclasts and has been shown to be a novel effective therapeutic target for osteoporosis. Zhang et al. focused on and summarized the advantages and disadvantages of the use of denosumab, the anti-RANKL antibody, in the treatment of postmenopausal osteoporosis. Although denosumab decreases osteoclast-mediated bone resorption and turnover, adverse events have also been reported after treatment, including skin eczema, flatulence, cellulitis, and osteonecrosis of the jaw. Considering the potential side effects of long-term medication of denosumab, aptamer has shown advantages and low toxicity and was hypothesized to be a promising candidate for therapeutic drugs targeting RANKL to counteract osteoporosis. Therefore, the determination of the three-dimensional structure of the RANKL-aptamer complex is necessary to discover the accurate binding domains and could be a crucial research basis for a functional aptamer targeting RANKL for the treatment of osteoporosis.

Protein kinase C delta (PKC-δ) functions as an important regulator in bone metabolism. Rong et al. conducted an osteoclast-specific PKC-δ knockout mouse strain to explore the function of PKC-δ in osteoclast biology. They found that ablation of PKC-δ in osteoclasts resulted in an increased bone volume in male mice, accompanied by decreased Cathepsin-K protein levels, osteoclast number, osteoclast formation, and resorption, whereas these changes were not observed in female mice. The work of this study revealed a previously unknown target for the treatment of gender-related bone diseases. In these two studies, they summarized the significance of RANKL-aptamer and PKC-δ and discuss the potential therapeutic strategies of the targeted modulation of the RANK/RANKL system and PKC-δ for bone-related diseases.

Taken together, increasing regulators have been revealed to participate in bone remodeling, which facilitates the mechanistic understanding of bone diseases and associated complications caused by disruption of bone remodeling, and provides new strategies for the treatment of bone diseases.

## Author Contributions

All authors listed have made a substantial, direct and intellectual contribution to the work, and approved it for publication.

## Funding

This work was supported by National Key R&D Program of China (Grant No. 2019YFA0111900), the National Natural Science Foundation of China (Grant Nos. 81922017, 81873669, 81802209, 81930022, 91749105, 81520108008, and 81873670), Hunan Provincial Science and Technology Department (2018RS3030), and the Australian National Health and Medical Research Council (NHMRC, APP1107828, APP1127156, and APP1163933).

## Conflict of Interest

The authors declare that the research was conducted in the absence of any commercial or financial relationships that could be construed as a potential conflict of interest.

## Publisher's Note

All claims expressed in this article are solely those of the authors and do not necessarily represent those of their affiliated organizations, or those of the publisher, the editors and the reviewers. Any product that may be evaluated in this article, or claim that may be made by its manufacturer, is not guaranteed or endorsed by the publisher.
